# A proof-of-concept study for the pathogenetic role of enhancer hypomethylation of *MYBPHL* in multiple myeloma

**DOI:** 10.1038/s41598-021-86473-y

**Published:** 2021-03-26

**Authors:** Kwan Yeung Wong, Gareth J. Morgan, Eileen M. Boyle, Alfred Sze Lok Cheng, Kevin Yuk-Lap Yip, Chor Sang Chim

**Affiliations:** 1 Department of Medicine, Queen Mary Hospital, The University of Hong Kong, Pokfulam Road, Pokfulam, Hong Kong; 2 NYU Langone Health, New York, NY 10016 USA; 3 School of Biomedical Sciences, The Chinese University of Hong Kong, Shatin, New Territories Hong Kong; 4 Department of Computer Science and Engineering, The Chinese University of Hong Kong, Shatin, New Territories Hong Kong

**Keywords:** Epigenetics, Myeloma

## Abstract

Enhancer DNA methylation and expression of *MYBPHL* was studied in multiple myeloma (MM). By bisulfite genomic sequencing, among the three CpGs inside the *MYBPHL* enhancer, CpG1 was significantly hypomethylated in MM cell lines (6.7–50.0%) than normal plasma cells (37.5–75.0%) (P = 0.007), which was negatively correlated with qPCR-measured *MYBPHL* expression. In RPMI-8226 and WL-2 cells, bearing the highest CpG1 methylation, 5-azadC caused enhancer demethylation and expression of *MYBPHL*. In primary samples, higher CpG1 methylation was associated with lower *MYBPHL* expression. By luciferase assay, luciferase activity was enhanced by *MYBPHL* enhancer compared with empty vector control, but reduced by site-directed mutagenesis of each CpG. RNA-seq data of newly diagnosed MM patients showed that *MYBPHL* expression was associated with t(11;14). MOLP-8 cells carrying t(11;14) express the highest levels of *MYBPHL*, and its knockdown reduced cellular proliferation and increased cell death. Herein, as a proof-of-concept, our data demonstrated that the *MYBPHL* enhancer, particularly CpG1, was hypomethylated and associated with increased *MYBPHL* expression in MM, which was implicated in myelomagenesis.

## Introduction

Multiple myeloma (MM) is characterized by the neoplastic proliferation of clonal plasma cells in the bone marrow^1,2^. Clinically, MM is defined by the presence of ≥ 10% clonal bone marrow plasma cells associated with features of end-organ damages including hypercalcaemia, renal failure, anaemia, or bone lesions that known as CRAB. These criteria have recently been updated to include the presence of any one of the following biomarkers, clonal bone marrow plasma cells of > 60%, a serum free light chain (SFLC) ratio of ≥ 100, or > 1 focal lesion in magnetic resonance imaging (MRI) studies^2^. Genetically, MM is a heterogeneous and can be broadly classified into non-hyperdiploid (NHRD) and hyperdiploid (HRD)^3,4^. NHRD is characterized by a primary translocations involving juxtaposition of the strong immunoglobulin heavy (IgH) chain at 14q32 upregulating one of a number of partner oncogenes including CCND1, FGFR3, MMSET, MAF, CCND3 and MAFB^3,4^. HRD in contrast is characterized by trisomies of odd-numbered chromosomes^3,4^. Moreover, both NHRD and HRD may carry secondary translocations involving MYC and other genetic abnormalities, including del(17p), del(13), amp(1q), del(1p), and RAS mutations^3,4^.

The effective regulation of gene control is essential for the specialized function of individual cells within a multicellular organism. DNA methylation is an important component of this process, which is frequently impacted in cancer^5,6^. A typical feature of methylation in carcinogenesis is global DNA hypomethylation associated with locus-specific hypermethylation at the promoter region of tumour suppressor genes^7,8^, features which are also typical of MM. Promoter DNA hypermethylation is associated with the recruitment of histone methyltransferases and histone deacetylases, followed by formation of a compact chromatin configuration leading to gene silencing and such processes are active in MM where methylation of promoter-associated CpG island has been shown to result in the inactivation of the tumour suppressors *SHP1* and *miR-34b/c*^9,10^.

A further element of gene control is provided by a class of *cis*-acting DNA sequence termed enhancers that are recognized by combinations of histone modifications, transcription factor occupancy, chromatin accessibility, and enhancer RNA expression^11–14^. Emerging evidence has demonstrated that structural genomic changes such as translocation, indel, and mutation, in enhancer regions could result in dysregulated gene expressions and hence disease pathogenesis^15^. Mechanistically, enhancer DNA is brought into close proximity with its target promoter region via DNA looping mediated by binding with transcription factors, mediator proteins, and activator proteins, resulting in formation of pre-initiation complex for gene transcription^16^. Major markers include the presence of H3K27ac and H3K4me1 but absence of H3K4me3^17,18^. Recently, enhancer DNA methylation has been implicated in the regulation of gene expression^18,19^. For example, by integrated analysis of enhancer regions, Illumina 450 K methylation array, and RNA-seq data, methylation of multiple enhancers, including the *MYBPHL* (myosin binding protein H like) enhancer, were shown to be inversely correlated with the expressions of their target genes in hepatocellular carcinoma (HCC)^18^.

*MYBPHL* encodes a protein with two immunoglobulin superfamily domains and a fibronectin 3 domain, the normal expression of which is restricted to the heart, however, there have been report of decreased methylation and increased expression in pituitary adenoma^20^. It is located at chromosome 1p13 a region frequently affected by structural variation and copy number change in MM^21^. Using gene expression analysis we noted that it is expressed in a subgroup of samples where it is associated with t(11;14). These observations suggest that it may be expressed as part of the malignant process as a consequence of methylation change. We have previously elucidated the role of methylation based control of the expression of this gene in hepatocellular cancer and we hypothesized it may also be important for malignant transformation of plasma cells. Herein, enhancer DNA methylation and expression of *MYBPHL* was studied in CD138-sorted normal plasma cells, human MM cell lines (HMCLs), and CD138-sorted primary samples of MM. Moreover, to the best of our knowledge, the data presented here is the first to demonstrate a potential oncogenic function of *MYBPHL* in MM.

## Results

### Enhancer of *MYBPHL* is methylated in normal plasma cells but hypomethylated in HMCLs

**Figure 1:**
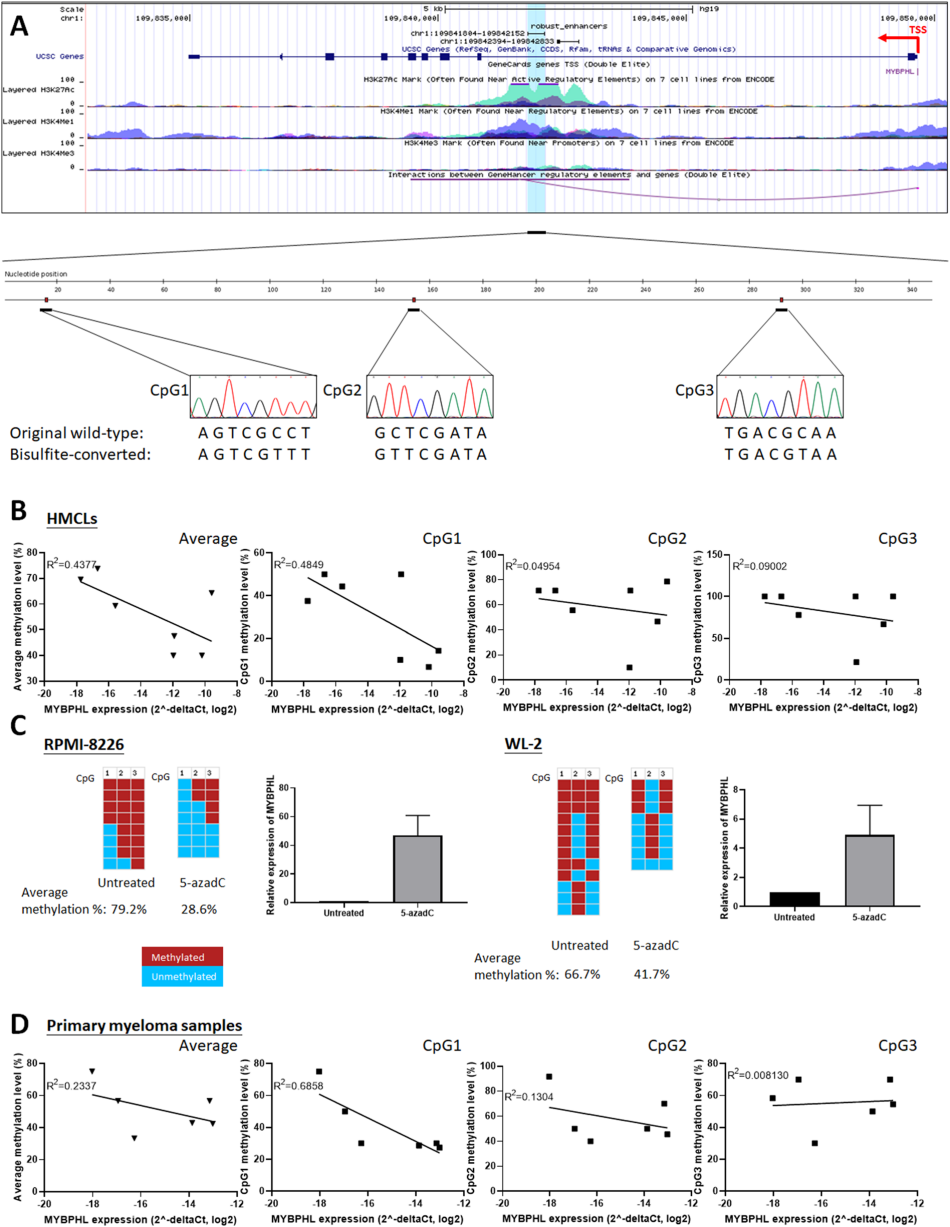
*MYBPHL* enhancer methylation and *MYBPHL* expression. (**a**) Schematic diagram of *MYBPHL* enhancer. *MYBPHL* enhancer (chr1:109841804–109842152), localized to 1p13, was retrieved from FANTOM5 Human Enhancer Tracks (http://slidebase.binf.ku.dk/human_enhancers/) and viewed in the UCSC Genome Browser^39^. Transcription start site (TSS) and the direction of transcription of *MYBPHL* were indicated by the red elbow arrow. *MYBPHL* enhancer region was shown overlapping with histone modifications annotating functional enhancer, i.e. presence of H3K27ac and H3K4me1 but absence of H3K4me3. Data from GeneHancer predicted interaction (downward curve) between *MYBPHL* enhancer to its target *MYBPHL* promoter^40^. Distribution of the three CpG dinucleotides on *MYBPHL* enhancer was depicted using BISMA ^41^. Bisulfite genomic sequencing (BGS) of the methylated positive control DNA showed conversion of all unmethylated cytosine residues into uracils (turned into thymidines after PCR), whereas methylated cytosine residues in CpG dinucleotides remained unchanged, indicating complete bisulfite conversion of DNA samples and specificity of BGS. (**b**) In HMCLs, methylation of all three CpG dinucleotides on average or in individual was plotted against *MYBPHL* expression as detected by qPCR. Data demonstrated that higher *MYBPHL* enhancer methylation, particularly CpG1, correlated with lower *MYBPHL* expression. (**c**) In RPMI-8226 and WL-2 cells, which had high level of *MYBPHL* enhancer methylation, treatment with 5-azadC led to *MYBPHL* enhancer demethylation, as evidenced by BGS, with concomitant re-expression of *MYBPHL* transcript, as detected by qPCR. (**d**) In primary myeloma samples, higher methylation of *MYBPHL* enhancer, particularly CpG1, correlated with lower *MYBPHL* expression. Data of qPCR were mean delta Ct values from three independent experiments with triplicate in each.

The enhancer DNA of *MYBPHL* has a genomic size of 348 bp and is embedded in the first intron of *MYBPHL,* which is localized at 1p13, and has been shown to be associated with three CpG dinucleotides, Fig. 1a. By bisulfite genomic sequencing (BGS), DNA methylation of the *MYBPHL* enhancer was studied in bisulfite-converted DNA of CD138-sorted normal plasma cells from healthy bone marrow donors (n = 7), human MM cell lines (HMCLs; n = 7), and an enzymatically methylated positive control DNA. The result from methylated positive control DNA showed conversion of all unmethylated non-CpG cytosine residues into uracils (turned into thymidines after PCR), whereas methylated cytosine residues in CpG dinucleotides remained unchanged, indicating complete bisulfite conversion of DNA samples and specificity of BGS, Fig. 1a. Of the three CpG dinucleotides in the *MYBPHL* enhancer, the mean methylation levels were 64.9% and 56.4% in CD138-sorted normal plasma cells and HMCLs, respectively, Table 1. Particularly, methylation of the first CpG dinucleotide (CpG1) was significantly higher in CD138-sorted normal plasma cells than HMCLs (58.8% vs. 30.4%; P = 0.007). These data suggest that the *MYBPHL* enhancer was hypomethylated, especially at CpG1, in HMCLs than compared to CD138-sorted normal plasma cells.

**Table 1:** Methylation of *MYBPHL* enhancer in normal plasma cells and myeloma cell lines (HMCLs were ordered according to the average methylation percentage).

Sample	Type	Number of clones	CpG1 (%)	CpG2 (%)	CpG3 (%)	Average (%)
PC	Positive control	10	100	100	100	100
N138-1	CD138-sorted normal plasma cells	12	75	58.3	75	69.4
N138-2	CD138-sorted normal plasma cells	9	55.6	100	88.9	81.5
N138-3	CD138-sorted normal plasma cells	6	66.7	83.3	50.0	66.7
N138-4	CD138-sorted normal plasma cells	10	60.0	50.0	60.0	56.7
N138-5	CD138-sorted normal plasma cells	9	66.7	66.7	55.6	63.0
N138-6	CD138-sorted normal plasma cells	8	37.5	50.0	62.5	50.0
N138-7	CD138-sorted normal plasma cells	2	50.0	100	50.0	66.7
RPMI-8226	HMCL	14	50.0	71.4	100	*73.8*
LP-1	HMCL	14	37.5	71.4	100	*69.6*
MOLP-8	HMCL	14	14.3	78.6	100	*64.3*
NCI-H929	HMCL	9	44.4	55.6	77.8	*59.3*
WL-2	HMCL	14	50.0	71.4	21.4	*47.6*
KMS-12-PE	HMCL	10	10.0	10.0	100	*40.0*
U-266	HMCL	15	6.7	46.7	66.7	*40.0*

*HMCL* human myeloma cell line, *N138* CD138-sorted normal plasma cells, *PC* positive control with methylated DNA.

Enhancer DNA methylation negatively correlated with expression of *MYBPHL.*

To investigate the relationship of enhancer DNA methylation and expression of *MYBPHL*, *MYBPHL* expression was measured in HMCLs (n = 7). A higher level of *MYBPHL* expression was demonstrated by Taqman-based qPCR, in HMCLs that were associated with lower *MYBPHL* enhancer methylation, Fig. 1b. However, among the three CpG dinucleotides, higher methylation of CpG1, but not CpG2 or CpG3, was demonstrated in HMCLs with lower *MYBPHL* expression. This result implicates this region as being the most potent region in controlling expression of this gene.

To validate the inverse relationship between enhancer DNA methylation and expression of *MYBPHL*, RPMI-8226 and WL-2 cells, which had highest CpG1 methylation, were treated with a hypomethylation agent, 5-azadC. Upon treatment with 5-azadC, both RPMI-8226 and WL-2 cells showed demethylation of the *MYBPHL* enhancer, as demonstrated by BGS, and this finding was associated with re-expression of *MYBPHL* transcripts by qPCR, Fig. 1c. In primary MM samples at relapse (n = 12), *MYBPHL* expression in CD138-sorted MM plasma cells was examined by qPCR. The result of this showed that *MYBPHL* enhancer methylation in six samples with paired CD138-sorted DNA available by BGS correlated with lower levels of expression of *MYBPHL*. Similarly, in primary samples, higher methylation of *MYBPHL* enhancer, especially CpG1 rather than CpG2 or CpG3, was correlated with lower *MYBPHL* expression, Fig. 1d.

**Figure 2:**
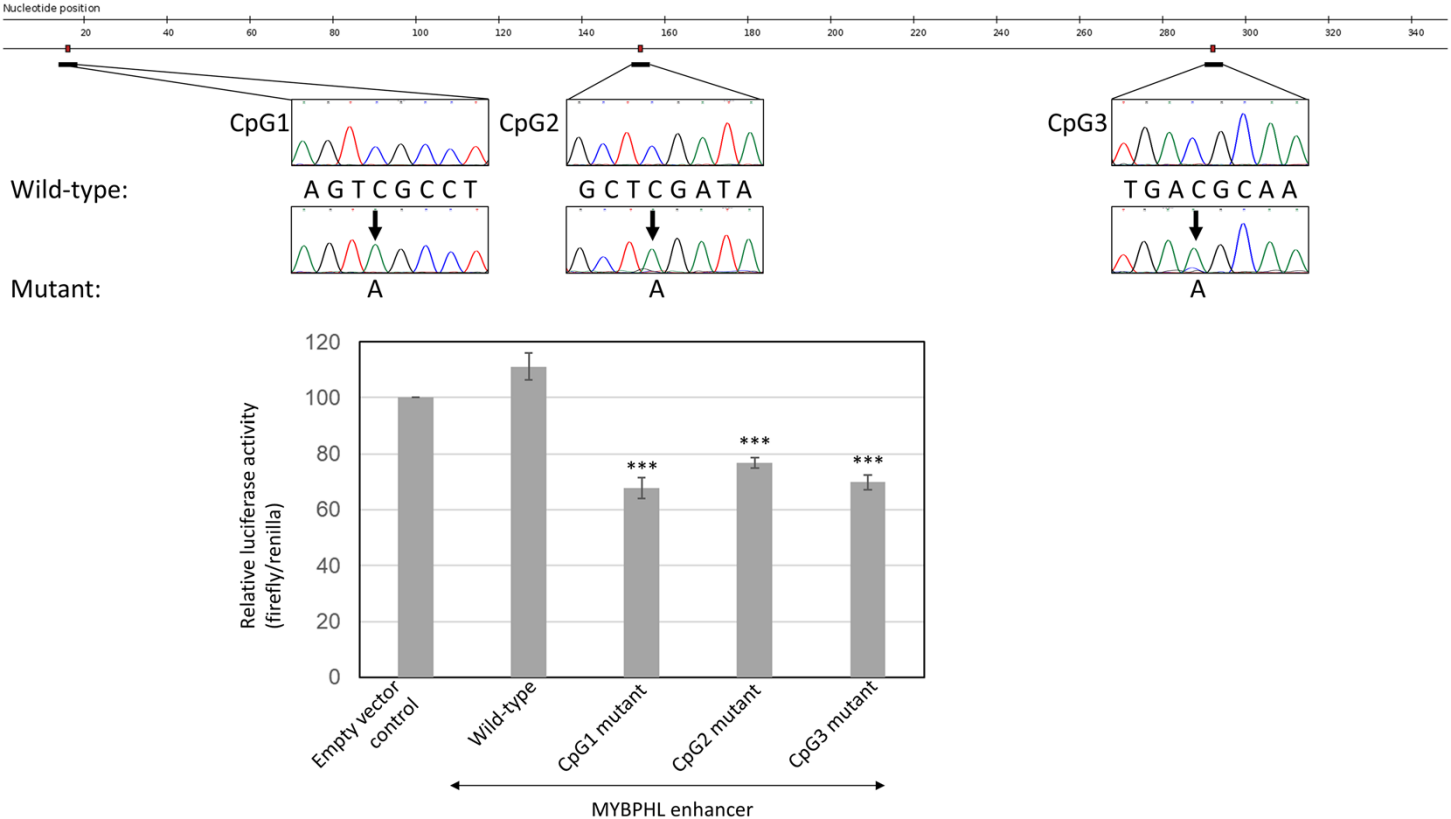
Enhancer-associated CpG dinucleotides and enhancer activity. By luciferase reporter assay, luciferase activity was increased by the presence of wild-type *MYBPHL* enhancer DNA, as compared with empty vector control. By site-directed mutagenesis of individual CpG into ApG, luciferase activity was significantly reduced, as compared with that of wild-type (***P ≤ 0.001). Data were mean ± s.d. of firefly/renilla from three independent experiments with triplicate in each.

To confirm the role of enhancer CpG dinucleotides on *MYBPHL* expression, the full-length *MYBPHL* enhancer was cloned and a comparison of constructs with site-directed mutagenesis at each of the three CpG dinucleotides by luciferase reporter assays was carried out. The results showed that luciferase activity was increased upon insertion of the *MYBPHL* enhancer in comparison with an empty vector control, demonstrating the transcriptional activity of the *MYBPHL* enhancer, Fig. 2. Moreover, by mutation of individual CpG into ApG, luciferase activity was significantly decreased as compared with wild-type *MYBPHL* enhancer, thereby functionally important for the *MYBPHL* enhancer on gene expression, Fig. 2.

*MYBPHL* is expressed in newly diagnosed MM patients.

**Figure 3:**
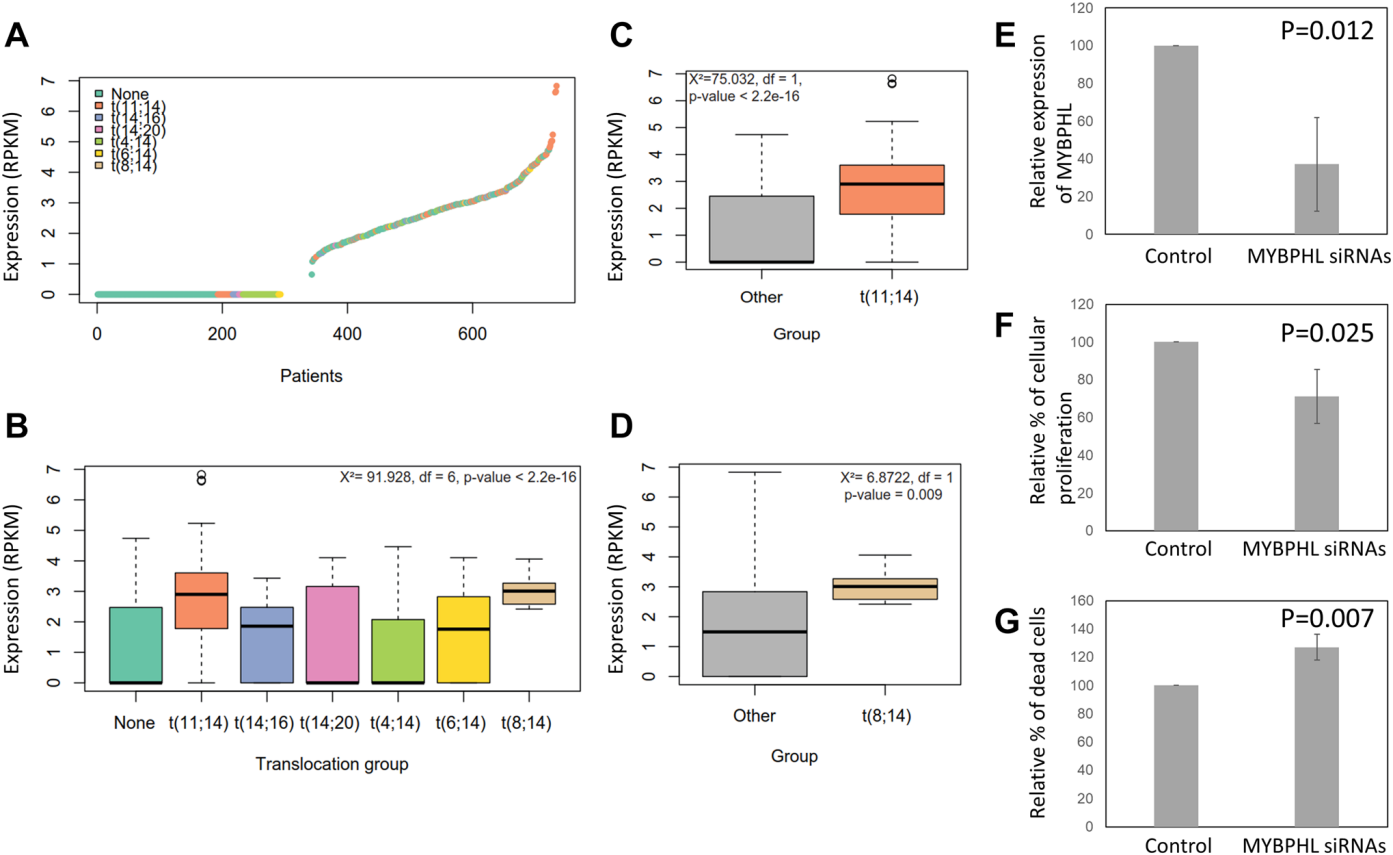
Expression and function of *MYBPHL* in MM cells. (**a**) Expression (RPKM) of *MYBPHL* by RNA-seq in 734 newly diagnosed MM patients. (**b**) Pattern of *MYBPHL* expression (RPKM) in different sequencing defined translocation subgroups. (**c**, **d**) Expression (RPKM) of *MYBPHL* is higher among the t(11;14) and t(8;14) patients. (**e**) Effect of *MYBPHL* knockdown in MM cells. In t(11;14)-bearing MOLP-8 cells, which had highest *MYBPHL* expression, *MYBPHL*-specific siRNAs were transfected, incubated for 48 h, followed by subsequent assays. By qPCR, *MYBPHL* expression was measured and compared with those transfected with scrambled negative control. (**f**) By MTS assay, cellular proliferation in response to *MYBPHL* knockdown was determined as compared with scrambled negative control. (**g**) By trypan blue staining, number of dead cells subjected to *MYBPHL* knockdown was examined as compared with scrambled negative control. Data were mean ± s.d. of three independent experiments with triplicate in each. *RPKM* reads per kilobase per million mapped reads.

Using the RNA-seq data from the CoMMpass study, we examined the expression of *MYBPHL* in newly diagnosed MM, and showed that it is either not expressed in 46% of patients (342/734) or expressed at low levels. However, in 9 of 734 cases, its level was expressed at greater than 5, Fig. 3a. Using, sequencing defined subgroups, we analyzed the overall expression of *MYBPHL* and showed that it varied among cytogenetic subgroups with higher expression levels seen in the CCND1 and MAFA groups [t(11;14) and t(8;14) respectively], Fig. 3b–d.

### *MYBPHL* is associated with oncogenic function in MM cells

The function of *MYBPHL* in MM cells was studied by siRNA-mediated knockdown. In MOLP-8 cells [carrying the t(11;14)(q13;q32)], in which *MYBPHL* expression was the highest among the HMCLs used, transfection with *MYBPHL*-specific siRNAs led to downregulation of *MYBPHL* expression by qPCR, Fig. 3e, or by Western blot, Supplementary Fig. 1, as compared cells transfected with scrambled negative control. By MTS assay, knockdown of *MYBPHL* led to inhibition of cellular proliferation by ~ 30%, Fig. 3f, as compared with scrambled negative control. Conversely, by trypan blue staining, knockdown of *MYBPHL* increased cell death by ~ 30%, Fig. 3g, as compared with scrambled negative control. Therefore, these data suggested a pro-proliferative role of *MYBPHL* in MM cells.

## Discussion

We explored a potential role for *MYBPHL* as an oncogene by siRNA knockdown in plasma cell lines overexpressing it. We demonstrated that knockdown of *MYBPHL* leads to decreased cellular proliferation and increased cell death, suggesting a potential novel role for *MYBPHL* in myelomagenesis in addition to its known functions in heart diseases^22,23^. We showed that *MYBPHL* is hypomethylated in MM cells in comparison with CD138-sorted normal plasma cells suggesting that it may function as an oncogene that is regulated by demethylation. Global DNA hypomethylation has been shown to be a hallmark of MM and increases as the disease progresses^24,25^. Such widespread DNA hypomethylation is linked to chromatin instability and transcriptional activation of oncogenes presumably via demethylation of promoter-associated CpG islands^26^. The *MYBPHL* enhancer is embedded in its first intron, about 7.5 kb downstream of the transcription start site. In the context of a potential oncogenic role for this gene, a recent genome-wide methylation study in MM has demonstrated that DNA hypermethylation is more likely to be associated with intronic enhancers than gene promoters and correlated with reduced enhancer activity and downregulation of their associated genes, e.g. *SLC15A4*, *PVT1*, and *NCOR2*^27^. Our data show that the expression of *MYBPHL* was repressed by hypermethylation at its intronic enhancer and hypomethylation was associated with upregulation.

Exploring the methylation based control of gene expression at this locus further we showed that different CpG dinucleotides at the same intronic enhancer had a differential influence in with the regulation of expression of the associated gene. Of the three CpG dinucleotides, CpG1 was mainly associated with the expression of *MYBPHL* in both HMCLs and primary samples of MM, therefore, these data not only rekindled the role of single-site CpG methylation in the regulation of gene expression, but also demonstrated that regulation by single-site CpG methylation was not restricted to the promoter region^28,29^. On the other hand, as the luciferase assay did not correspondingly indicate that C- > A mutation at CpG1 affected the enhancer activity more than CpG2 or CpG3, thereby suggesting that methylation of CpG1 might also affect something other than the enhancer activity, such as interaction with the target promoter. Even though enhancer landscapes are considered to be cell-specific^13,30^, we clearly showed that the interaction of the *MYBPHL* enhancer-promoter pair in myeloma plasma cells is consistent with the previous report in HCC^18^.

In MM, as emerging evidences have shown that, in contrast to normal cells, cancer cells were more sensitive to enhancer-selective inhibitors, such as inhibitors targeting BET-bromodomain protein 4 (BRD4) and cyclin-dependent kinase 7 (CDK7)^31–33^, these data further support to the concept of incorporating enhancer-targeting inhibitors in the treatment of MM. Moreover, by the advent of genome-wide technologies interrogating CpG dinucleotides, in particular those residing outside of promoter regions, which had not been covered before, our data indicate that further studies of DNA methylation at “nascent” CpG dinucleotides may give new insights into the regulation of gene expression in cancer and may provide either potential therapeutic interventions or the identification of methylation based biomarkers with clinical significance^34^.

Of note, these data could be strengthened by correlating *MYBPHL* methylation and expression in normal plasma cells. However, CD138-sorted normal plasma cells had been exhausted for DNA extraction, whereby a higher level of *MYBPHL* methylation was demonstrated in normal plasma cells than HMCLs (Table 1). In fact, in contrast to solid cancers, in which normal controls are readily available adjacent to tumour cells, normal plasma cells as a control for MM plasma cells are much more difficult to obtain. As normal plasma cells only constitute < 1% of mononuclear cells in the bone marrow, collection of sufficient CD138-sorted normal plasma cells for both DNA and RNA extraction is extremely difficult. Moreover, in the past, normal bone marrow plasma cells were mainly derived from bone marrow stem cells collected by bone marrow puncture of healthy donors under general anesthesia, whereby normal plasma cells could be isolated. In the last two decades, haematopoietic stem cells were instead collected by apheresis after mobilization of stem cells into the peripheral blood, hence precluding isolation of normal marrow plasma cells. In the future, similar to a recent publication in MM^35^, removed hip in patients receiving total hip replacement could serve as an alternative source of normal plasma cells for both DNA and RNA extraction. Lastly, the current study serves as a proof-of-principle pilot study, conducive to future enhancer methylation studies in MM that will include more primary samples.

## Methods

### Patient samples

Twelve patients with relapsed/refractory multiple myeloma (RRMM), including “relapsed” or “relapsed-and-refractory myeloma” cases were included for study of *MYBPHL* expression. Definition of relapsed myeloma is consistent with previously described^36^. This study was approved by the Institutional Review Board of Queen Mary Hospital. Written informed consent was obtained from all participants involved in the study. Normal bone marrow plasma cells were obtained from healthy marrow donors for bone marrow transplantation. All study methods were performed in accordance with relevant guidelines and regulations.

### Cell culture

Human MM cell lines (HMCLs) KMS-12-PE, MOLP-8, and U-266 were purchased from Deutsche Sammlung von Mikroorganismen und Zellkulturen (Braunschweig, Germany). NCI-H929 was purchased from American Type Culture Collection (Manassas, VA, USA). LP-1 and RPMI-8226 were kindly provided by Prof. Robert Orlowski (Department of Lymphoma/Myeloma, Division of Cancer Medicine, The University of Texas MD Anderson Cancer Center, Houston, TX, USA). WL-2 was kindly provided by Prof. Andrew Zannettino (Myeloma Research Programme, The University of Adelaide, Australia). Cell lines were cultured in RPMI-1640 medium (IMDM for LP-1), supplemented with 10% fetal bovine serum, 50 U/mL of penicillin and 50 μg/mL streptomycin, in a humidified atmosphere of 5% CO_2_ at 37 °C. All cell culture reagents were purchased from Thermo Fisher Scientific (Waltham, MA, USA).

### Bisulfite genomic sequencing

Bisulfite-treated DNA was used as template. Enhancer DNA of *MYBPHL* was amplified and cloned using TOPO TA Cloning Kit (Thermo Fisher Scientific), followed by sequencing, according to the manufacturer's instructions. Primers used were forward 5′-GGG GGT TTG GTT GAG ATA GAT AT-3′ and reverse 5′-ACC CAA ATA AAA ACA TAA ACC ACA C-3′ ^18^. PCR conditions for MgCl_2_/Tm/cycles were 2.0 mM/56 °C/35X. Methylated Human Control (Promega) was used as positive control for DNA methylation.

### Hypomethylation treatment

Cells were cultured and treated with a hypomethylating agent, 5-aza-2′-deoxycytidine (5-azadC; Sigma-Aldrich, St. Louis, MO, USA) as previously described^10^.

### Knockdown of MYBPHL

*MYBPHL*-specific siRNAs (Assay ID: s50998, s50999, s51000) and Cy3-labeled scrambled negative control siRNA (Cat. no.: AM4621) were purchased from Thermo Fisher Scientific (Waltham, MA, USA). Briefly, MOLP-8 cells were seeded at a density of 0.5 × 10^6^ cells per well in a 24-well plate and transfected with *MYBPHL*-specific siRNAs (25 nM) or scrambled negative control siRNA using Lipofectamine RNAiMAX (Invitrogen, Carlsbad, CA, USA). Cells were harvested at 48 h post-transfection, followed by functional assays.

### MTS assay

Cellular proliferation was measured by MTS assay (Promega; Madison, WI, USA). In brief, transfected cells of 5 × 10^4^ were resuspended in 100 µL medium and seeded in a 96-well microplate in triplicate. At 48 h post-transfection, MTS reagent of 20 µL was added into each well, followed by incubation for a further 4 h. Absorbance at 490 nm was measured using Epoch Microplate Spectrophotometer (BioTek; Winooski, VT, USA), background subtracted, and normalized to cells transfected with scrambled negative control siRNA. Means of three independent experiments with triplicate in each were plotted.

### Trypan blue staining

Cell death was examined by staining with trypan blue (Thermo Fisher Scientific). Briefly, at 48 h post-transfection, number of unstained live cells and stained dead cells were counted in five random microscopic fields under a microscope, followed by normalization to cells transfected with scrambled negative control siRNA. Means of three independent experiments were plotted.

### Luciferase reporter assay

Firefly luciferase reporter plasmid constructs (pGL3-Promoter Vector, Promega, Madison, WI, USA) cloned with enhancer DNA of *MYBPHL* (348 bp) were kind gifts from Dr. Alfred Sze Lok CHENG (School of Biomedical Sciences, CUHK)^18^. On the enhancer DNA of *MYBPHL*, each cytosine residue of the three CpG dinucleotides, located at nucleotide position 16, 154, and 292, was individually mutated into adenine residues using QuikChange Lightning Multi Site-Directed Mutagenesis Kit (Stratagene, La Jolla, CA, USA) as previously described^28^. Briefly, SW480 cells (kind gift from Dr. Roberta PANG, Department of Surgery, HKU) were seeded at a density of 2 × 10^5^ cells per well in a 24-well plate overnight, followed by co-transfection with firefly and renilla (pGL4.75[hRluc/CMV] Vector, Promega, Madison, WI, USA) luciferase reporter plasmids at a ratio of 500:0.75 using Lipofectamine 2000 (Invitrogen, Carlsbad, CA, USA). Cells were harvested at 24 h post-transfection. Firefly and renilla luciferase activity were sequentially generated using Dual-Luciferase Reporter Assay System (Promega, Madison, WI, USA), and their luminescent signals were measured using CLARIOstar microplate reader (BMG LABTECH, Cary, NC, USA). Luciferase activity of firefly was normalized by renilla and relative luciferase activity was obtained as compared with empty pGL3-Promoter Vector. Data represents mean of three independent transfections with triplicate in each.

### RNA-seq analysis

We analyzed RNA-seq from newly diagnosed MM (NDMM) samples from the CoMMpass dataset (n = 734). RNA-seq data that were uniformly processed. Salmon21 (v0.7.2) was used to align reads to the transcriptome and quantify expression at the gene and transcript level as previously published^37^. Expression levels were compared across cytogenetic groups amongst patients that had WES data available (n = 628)^38^.

### Statistical analysis

Methylation and expression of *MYBPHL* were plotted and analyzed using GraphPad Prism 8 (GraphPad Software; La Jolla, CA, USA). *MYBPHL* methylation between CD138-sorted normal plasma cells and HMCLs, data between cells transfected with *MYBPHL*-specific siRNAs and scrambled negative control siRNA were compared by Student’s *t*-test. All P values were two-sided, and P < 0.05 was defined as a significant difference.

## Supplementary Information

Supplementary Figure 1.

## Data Availability

All data generated or analysed during this study are included in this published article (and its Supplementary Information files).
