# Supplementary material for: A proof-of-concept study for the pathogenetic role of enhancer hypomethylation of MYBPHL in multiple myeloma
Source: Sci Rep. 2021 Mar 26;11:7009. doi: 10.1038/s41598-021-86473-y (PMC7997988; doi:10.1038/s41598-021-86473-y)

Supplementary Information

Article

A proof-of-concept study for the pathogenetic role of enhancer hypomethylation of *MYBPHL* in multiple myeloma

Kwan Yeung WONG[1], Gareth J. MORGAN[2], Eileen M. BOYLE[2], Alfred Sze Lok CHENG[3], Kevin Yuk-Lap YIP[4], Chor Sang CHIM[1#]

1 Department of Medicine, Queen Mary Hospital, The University of Hong Kong, Pokfulam Road, Pokfulam, Hong Kong;

2 NYU Langone Health, New York, NY 10016, USA;

3 School of Biomedical Sciences, The Chinese University of Hong Kong, Shatin, New Territories, Hong Kong;

4 Department of Computer Science and Engineering, The Chinese University of Hong Kong, Shatin, New Territories, Hong Kong.

#Corresponding author: Prof. Chor Sang CHIM, MD, PhD, Department of Medicine, Queen Mary Hospital, The University of Hong Kong, Pokfulam Road, Pokfulam, Hong Kong.

Email: jcschim@hku.hk

Tel: (852) 2255 4769

Fax: (852) 2816 2187

ORCID: 0000-0003-2427-915X

Running title

MYBPHL enhancer methylation in myeloma

Keywords

MYBPHL, enhancer, DNA methylation, multiple myeloma, t(11;14)

Supplementary Figure 1. Western blot analysis of MYBPHL in MM cells. In t(11;14)-bearing MOLP-8 cells, which had highest *MYBPHL* expression, *MYBPHL*-specific siRNAs were transfected, incubated for 48 hours. Western blots using antibodies against MYBPHL (Abcam, Cambridge, UK; ab197216) and β-ACTIN (Cell Signaling Technology, Danvers, MA, USA; 4970) were performed. MYBPHL expression in MOLP-8 cells transfected with *MYBPHL*-specific siRNAs was compared with those transfected with scrambled negative control siRNA. HeLa cells were used as positive control. Cropped blots were shown, whereas full-length blots are shown in Supplementary Figure 2.

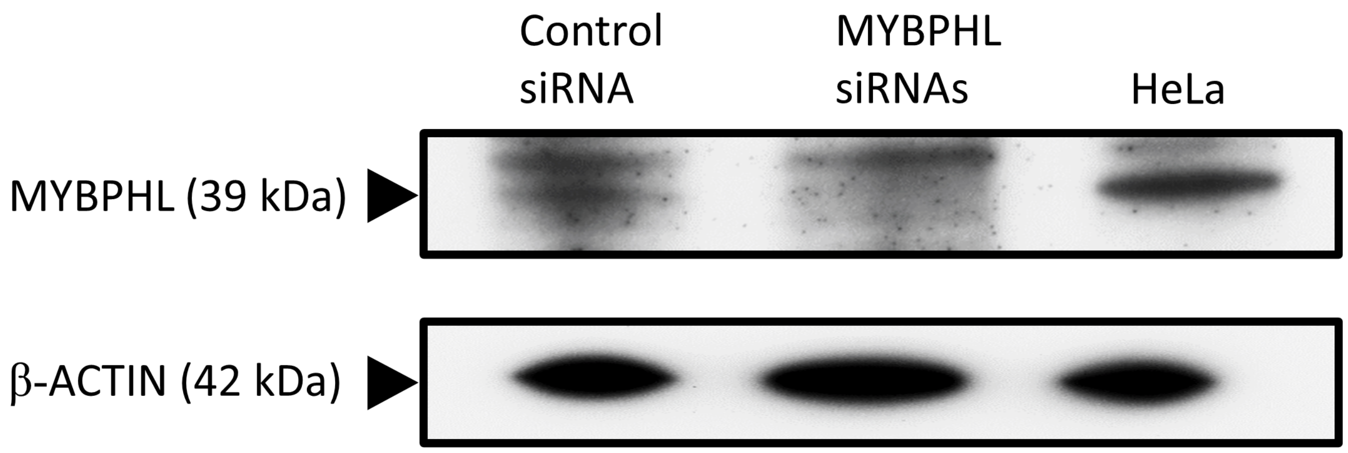

Supplementary Figure 2. Full-length blots of MYBPHL (39 kDa; top panel) and β-ACTIN (42 kDa; bottom panel).

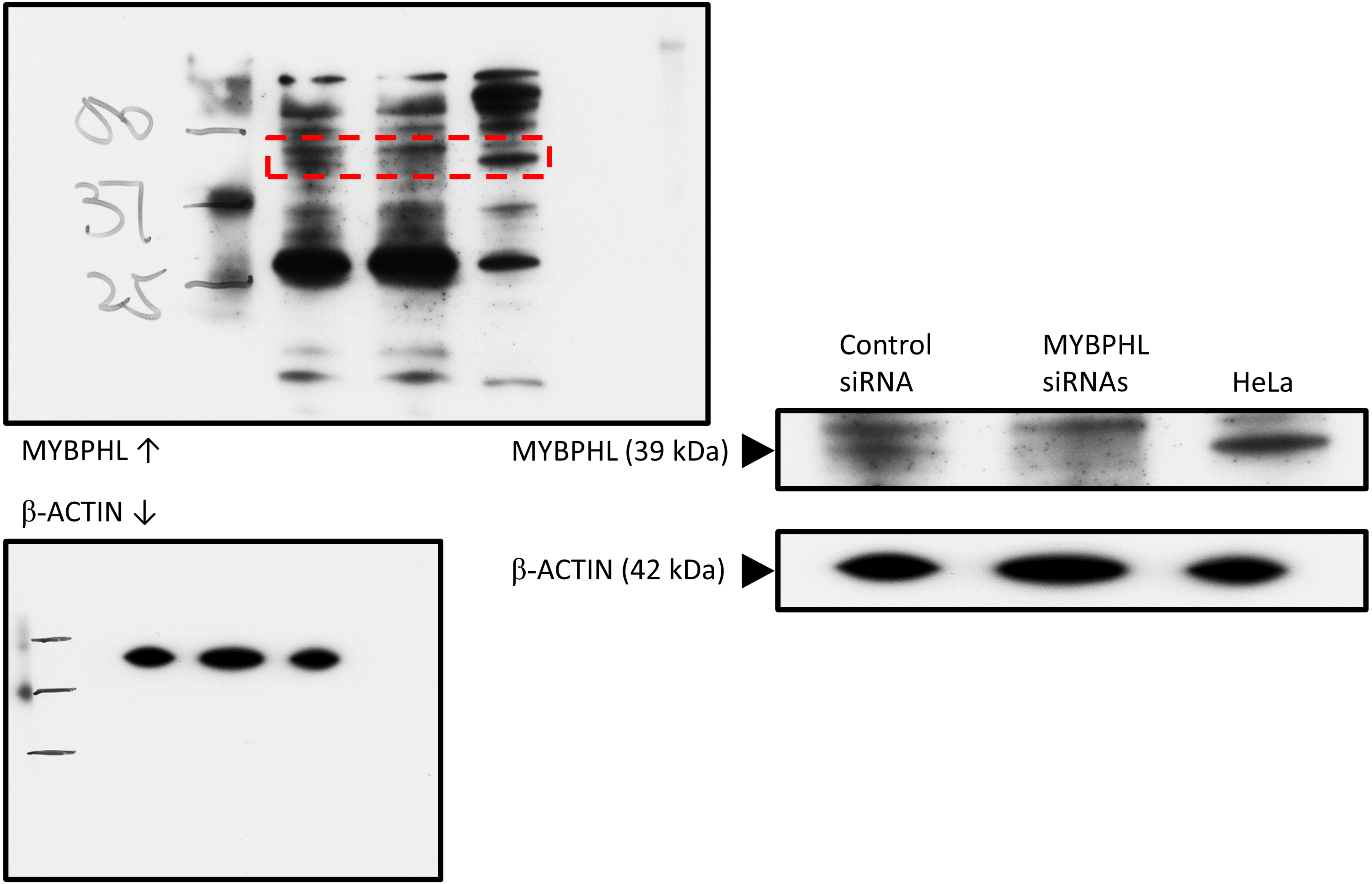

Supplement: Supplementary file 1 — Supplementary Figure 1. [file 41598_2021_86473_MOESM1_ESM.pdf]
